# Supplementary material for: The Rootstock Genotypes Determine Drought Tolerance by Regulating Aquaporin Expression at the Transcript Level and Phytohormone Balance
Source: Plants (Basel). 2023 Feb 6;12(4):718. doi: 10.3390/plants12040718 (PMC9961603; doi:10.3390/plants12040718)
Supplement: Supplementary file 1 [file plants-12-00718-s001.zip › plants-2146381-supplementary.pdf]

The rootstock genotypes determine drought tolerance by regulating aquaporin expression and phytohormone balance

David Labarga, Andreu Mairata, Miguel Puelles, Ignacio Martín, Alfonso Albacete, Enrique García-Escudero and Alicia Pou

SUPPLEMENTARY MATERIAL:

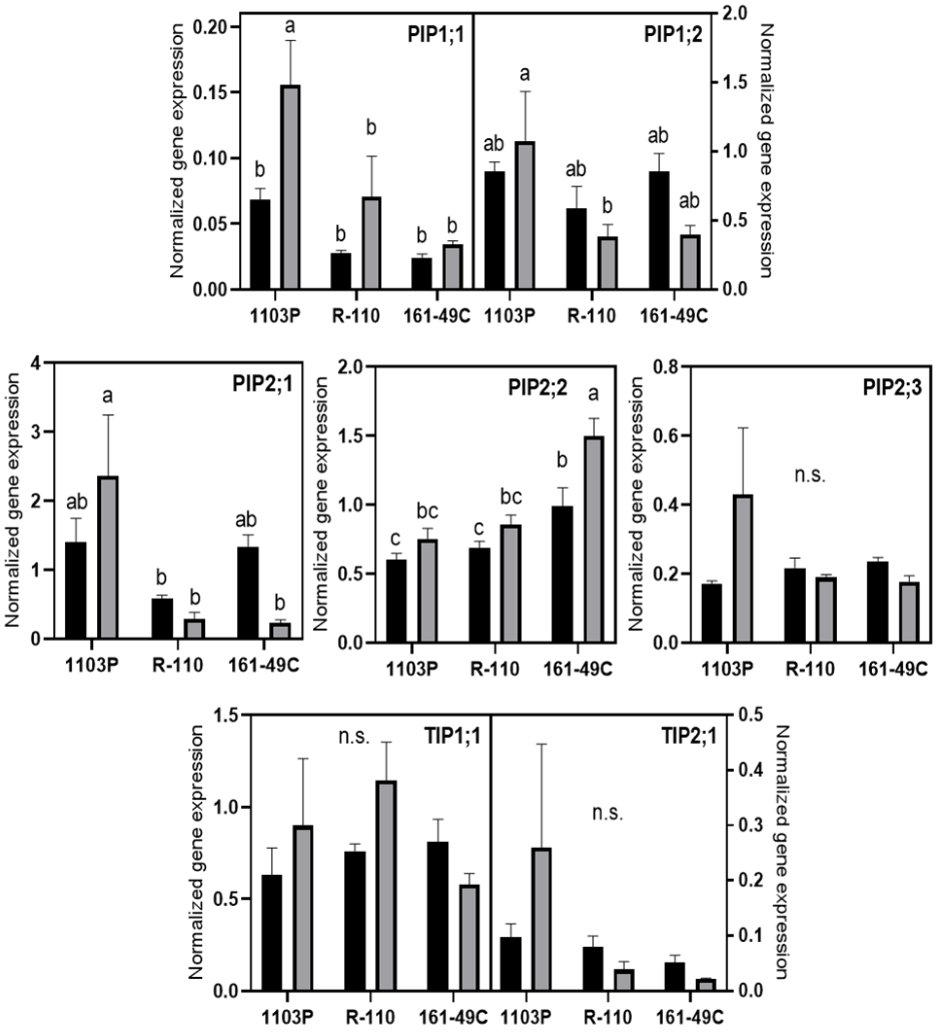

Figure S1: 2^ΔCt of studied aquaporin genes for each rootstock under well water (WW) (black) and water stress (WS) (grey). Different letters indicate statistically significant differences by one-way ANOVA with Duncan's multiple comparison test ($p<0.05$) for each aquaporin gen studied.

Table S1. Mean values of several vegetative growth components of the different rootstocks: clusters, pruning weight, spurs per vine, shoots per vine, 100 berries weight, total clusters weight and cluster weight.

		Clusters	Pruning weight (kg ha^{-1})	Spur (spur vine^{-1})	Shoots (shoots vine^{-1})	100 berries weight (g)	Total clusters weight (kg vine^{-1})	Cluster weight (g cluster^{-1})
1103P	Irrigation	17.40 ± 7.02	2592	5,80 ± 0,84	13.00 ± 1.73	220.30	3.65	209.57
	Drought	15.67 ± 5.24	1173	7.00 ± 1.58	14.20 ± 4.38	185.90	3.57	227.66
R-110	Irrigation	19.33 ± 1.51	2089	6.00 ± 0.71	13.80 ± 2.17	226.40	3.70	191.38
	Drought	16.83 ± 3.60	821	5.60 ± 1.14	11.80 ± 1.79	191.80	2.65	157.43
161-49C	Irrigation	16.50 ±5.65	1790	6.40 ± 0.89	14.00 ± 2.12	239.70	3.48	211.11
	Drought	16.17 ± 5.15	562	5.80 ± 1.30	12.60 ± 3.21	161.30	2.18	135.05
Significance	TR	**n.s.**	-	**n.s.**	**n.s.**	-	-	-
	RT	**n.s.**	-	**n.s.**	**n.s.**	-	-	-
	TR x RT	**n.s.**	-	**n.s.**	**n.s.**	-	-	-

Values are means ± standard error of six vines per treatment. Significant differences for treatment (TR), rootstock (RT) and TR × RT were analyzed by two-way ANOVA (ns, not significant; *, p ≤ 0.05; **, p ≤ 0.01; ***, p ≤ 0.001). The bar (-) indicates there were not enough replicates to perform statistical analysis.

Table S2. Primer sequences of each aquaporin gene studied as well as the reference where the sequences were obtained.

Vitis gene		Primer	Reference
**PIP 1.1**	Forward:	5'-GAGTGGTGCTGGGCGTTGATC-3'	Gambetta et al. [109]
	Reverse:	5'-GTGGAATGCTACAGACATTAC-3'	
PIP 1.2	Forward:	5'-CGCCATCGTCTACAACAAAG-3'	Vandeleur et al. [38]
	Reverse:	5'-CAGGCTCTGGTCTTGAATGG-3'	
PIP 2.1	Forward:	5′-GGCATTTCTGGGGGACACAT-3′	Dayer et al. [40]
	Reverse:	5′-CTTTGACGAGACCCACACCA-3′	
PIP 2.2	Forward:	5'-AACTAAAAACCCACAACACCC-3'	Gambetta et al. [109]
	Reverse:	5'-CATCATCATAATCATCTCTGG-3'	
PIP 2.3	Forward:	5′- GCCATTGCAGCATTCTATCA-3′	Pou et al. [30]
	Reverse:	5′-TCCTACAGGGCCACAAATTC-3′	
TIP 1.1	Forward:	5'-GTTGTTGTCTCAACCCATTTCC-3'	Galmés et al. [72]
	Reverse:	5'-ATCACCAACCTCATTCATATGC-3'	
TIP 2.1	Forward:	5′-GGAGGAAGAGCAAGTTGTGC-3′	Pou et al. [30]
	Reverse:	5′-GCACATCACCAACCTCATTC-3′	
Ubiquitin	Forward:	5′-GTGCTGTCAACTGCAGGAAA-3′	Pou et al. [30]
	Reverse:	5′-GTAGCCATGGCACATCCAAT-3′	
ELF	Forward:	5′-CGGGCAAGAGATACCTCAAT-3′	Dayer et al. [40]
	Reverse:	5′-AGAGCCTCTCCCTCAAAAGG-3′	
Actin	Forward:	5'- GCCTCCGATTCTCTCTGCTCTC -3'	Vandeleur et al. [76]
	Reverse:	5'- TCACCATTCCAGTTCCATTGTCAC -3'	
